# Thompson’s Compound Self-Adjusting Blow Pipe

**Published:** 1853-07

**Authors:** 


### THOMPSON'S COMPOUND SELF ADJUSTING BLOW PIPE.

This we regard as one of the most perfect improvements which has been introduced for rendering the operation of soldering easy and pleasant. This instrument consists of a Bellows which sets on the floor, and is worked by the foot, to which is attached a flexible tube, which passes up through a lamp containing alcohol. This has a handle attached, and is held in the hand, and with the thumb the lever is worked, which lets on the current of air supplied by the working of the Bellows. The jet is perfectly under the control of the operator, and the instrument throughout is one of the most simple and complete we have seen.

